# Reflections on one year in the Americas: What is next for the region?

**DOI:** 10.1016/j.lana.2022.100370

**Published:** 2022-09-14

**Authors:** The Lancet Regional Health– Americas

In our inaugural Editorial, we pledged to be a strong platform to raise awareness, stimulate public debate about regional health issues, and help advance locally relevant research and health-care provision. In this 1-year anniversary Editorial, we reflect on the journal's past contributions and the upcoming challenges for the region.

Over the past year, we have published 171 research articles and over 97 opinion pieces covering many pressing topics for the community we vouched to serve. The journal was launched amidst the COVID-19 pandemic and published randomised trials and large cohort studies on COVID-19 risks, treatments, and vaccine safety and efficacy. Most vaccine studies were conducted in Argentina, Brazil, Colombia, Cuba, and the USA, contributing to the global knowledge and fostering evidence-based decision-making for policies. Vaccination, however, has been more challenging than expected, as inequity in distribution has prevented and delayed access, particularly in South America and the Caribbean. Barberia and colleagues exposed a deficit of over 7 million doses required to vaccinate children aged 3–5 years in Brazil, a result from the systematic delay in vaccine approval and distribution for young children in the country. The Comment received national coverage and, although the purchase of additional doses was announced by the Brazilian Government, the response is vastly insufficient and would still leave millions of children excluded from immunisation.

The politization of science, meaning the influence of politics and politician views in how the general population perceives and believes (or not) in science, is a growing threat in several countries in America—including the USA and Brazil, which are the world's and South America's largest economies, respectively. A retrospective study by Xavier and colleagues revealed a positive association between increased COVID-19 mortality rates and support for the far-right President Jair Bolsonaro at the municipality level in Brazil. Similarly, lower vaccine coverage was observed in US states with higher support for former President Donald Trump. Both leaders, Trump and Bolsonaro, have repeatedly disseminated misinformation regarding COVID-19 treatments and fuelled vaccine hesitancy during the COVID-19 pandemic, contributing to the distrust in science and scientific-based decisions.

The consequences of the politization of science go beyond the disastrous management of the COVID-19 pandemic. In June, 2022, when the US Supreme Court decided to overturn Roe *v* Wade, leaving the legal right to abortion decision to each of the US states, we published a study from Mikaela Smith and collaborators describing the reality of abortion access for people who lived in the US states with restrictive abortion legislation. The devastating impacts of turning abortion rights into a political decision, and the threat to women's health and safety was discussed in an accompanying Editorial.

In a powerful opinion piece, Aranda and colleagues called for culturally sensitive COVID-19 immunisation campaigns targeted to Indigenous people of Latin America and the Caribbean, respecting their rights to autonomy and self-determination. Historically excluded, Indigenous people's voices have been silenced in various territories in the Americas, threatening their health and safety. The escalating violence against Indigenous leaders and activists in the Amazon basin and the systematic disrespect to their territorial sovereign and rights—mainly driven by political interests to explore the natural resources and use the land for agricultural business, mining, and oil drilling—was highlighted in our July 2022 Editorial.

A direct consequence of irresponsible exploration of resources, the impact of climate crises is being felt throughout the Americas, yet most countries still lag behind in their climate action agendas. Following the release of the 2021 Lancet Countdown report, Yglesias-González and colleagues discussed the climate and health challenges in Latin American and the Caribbean and called for bold, fast, and equitable climate action to protect people's health in the region. Housing a significant part of the world's largest carbon sink, the Amazon Forest, Brazil is a key player for reducing the impact of climate change on health. As the country gets ready to elect a new president this coming October, there is hope that the new government will reinstate the country's commitment to halt deforestation, increase protection of the Amazon biome and Indigenous people, and prioritise climate mitigation plans in its political agenda.

It has been quite a year for the Americas, and much more remains for the months and years to come. Directing the health workforce to COVID-19 pandemic efforts deeply affected prevention and continuous-care programmes, with disruptions to the HIV cascade-of-care documented for both North and Latin America and the Caribbean. Peru and Brazil are among the top 16 contributors to the global shortfall in tuberculosis notifications in 2020; while Peru also registered a 34% increase in dengue cases in 2021, prompting an emergency declaration. The COVID-19 pandemic has left a trail of destruction, and the consequences of 2 years of discontinued health-care provision associated with the global economic crisis will be felt hard in the region. The importance of universal health-care access and resilient health-care systems is clearer than ever, and investments to reformulate and rebuild how we provide health care should be a priority for local leaders. Rebuilding our region will require multisectoral efforts to address the many challenges to come, including the educational gap created by school closures, the reduced vaccination coverage, and the re-emergence of diseases. The PAHO has a historically strong participation in the region and, as the organisation moves to elect its new president by the end of this month, a bold and ambitious plan to revert the COVID-19 effects must be a top priority in the candidates´ agenda. As we walk towards recovery, it is imperative that countries and governing bodies put population health in the centre of their agendas and use evidence-based decisions, prioritising investment over profit.

As a journal, we will continue to build an open-access platform for research scientists, clinicians, and policy makers in the region for constructive discussions that can help advance locally relevant research and health care. We will continue to advocate for and support all research that promotes advancements in health and policy for historically marginalised populations and helps our society build an equal, sustainable future.

